# Fabrication and Characterization of Electrospun Cu-Doped TiO_2_ Nanofibers and Enhancement of Photocatalytic Performance Depending on Cu Content and Electron Beam Irradiation

**DOI:** 10.3390/polym16050694

**Published:** 2024-03-04

**Authors:** So-Hyeon Lee, Kyeong-Han Na, Jae-Yoon Kim, Han-Sol Yoon, HyukSu Han, Won-Youl Choi

**Affiliations:** 1 Department of Advanced Materials Engineering, Gangneung-Wonju National University, 7 Jukheongil, Gangneung 25457, Republic of Korea; thtth@naver.com (S.-H.L.); paul8060@gwnu.ac.kr (J.-Y.K.); ac.hsyoon@gmail.com (H.-S.Y.); 2 Smart Hydrogen Energy Center, Gangneung-Wonju National University, 7 Jukheongil, Gangneung 25457, Republic of Korea; nag0717@gwnu.ac.kr; 3 Research Institute for Dental Engineering, Gangneung-Wonju National University, 7 Jukheongil, Gangneung 25457, Republic of Korea; 4 Department of Energy Science, Sungkyunkwan University, Suwon 16419, Republic of Korea; hyuksuhan@skku.edu

**Keywords:** TiO_2_ nanofibers, Cu doping, electron beam irradiation, photocatalysis

## Abstract

Titanium dioxide (TiO₂) is a widely studied material with many attractive properties such as its photocatalytic features. However, its commercial use is limited due to issues such as deactivation in the visible spectrum caused by its wide bandgap and the short lifetime of photo-excited charge carriers. To overcome these challenges, various modifications could be considered. In this study, we investigated copper doping and electron beam treatment. As-spun TiO_2_ nanofibers were fabricated by electrospinning a TiO_2_ sol, which obtained viscosity through a polyvinylpyrrolidone (PVP) matrix. Cu-doped TiO_2_ nanofibers with varying dopant concentrations were synthesized by adding copper salts. Then, the as-spun nanofibers were calcined for crystallization. To evaluate photocatalytic performance, a photodegradation test of methylene blue aqueous solution was performed for 6 h. Methylene blue concentration was measured over time using UV-Vis spectroscopy. The results showed that Cu doping at an appropriate concentration and electron-beam irradiation showed improved photocatalytic efficiency compared to bare TiO_2_ nanofibers. When the molar ratio of Cu/Ti was 0.05%, photodegradation rate was highest, which was 10.39% higher than that of bare TiO_2_. As a result of additional electron-beam treatment of this sample, photocatalytic efficiency improved up to 8.93% compared to samples without electron-beam treatment.

## 1. Introduction

In modern society, the emission of environmental pollutants such as waste, wastewater, and smoke are increasing due to population growth and industrial development [1,2,3]. As solutions to these issues are demanded, interest in eco-friendly and effective water treatment systems is increasing [4,5]. There are various water treatment methods utilizing mechanisms such as filtration, coagulation, centrifugation, and flocculation [6,7,8,9]. Among these, photocatalytic degradation, which harnesses renewable energy, has attracted attention for its efficient cost-effectiveness and environmentally friendly advantages [10,11,12]. Due to their cost efficiency and durability, transition metal-based semiconductors like TiO_2_, CdS, ZrO_2_, ZnO, and WO_3_ are commonly discussed as representative photocatalyst materials [13,14,15,16]. When semiconductor photocatalysts are exposed to light with a wavelength equal to or smaller than their energy bandgap, electrons in the valence band become excited to the conduction band, forming electron–hole pairs. As these charge carriers diffuse to the surface of the photocatalyst, they react with water and then generate free radicals such as superoxide (•O_2_^−^) or hydroxyl radicals (•OH). These radicals contact pollutants in water, resulting in the decomposition of molecular structures [16,17]. Since the demonstration of water splitting using ultraviolet light by Honda and Fujishima in 1972, TiO_2_ has become the most widely researched photocatalytic material [18,19,20]. Due to its low cost, high chemical stability, and non-toxic properties, TiO_2_ is a suitable material for photocatalytic applications [21,22,23]. However, the wide bandgap of 3.2 eV restricts its reaction in the visible light spectrum, and the rapid recombination of photoexcited electron–hole pairs poses a challenge for applying TiO_2_ as a photocatalyst [24,25]. To overcome the challenges associated with TiO_2_ photocatalysts, many studies have applied methods such as impurity doping, heterojunction constructions, surface/microstructure modification, and sensitization [26,27,28,29,30,31,32]. However, it is difficult to find studies that irradiate electron beams on Cu-doped TiO_2_ nanofibers. In this study, TiO_2_ nanofibers (TNFs) were fabricated using an electrospinning process, and the effects of copper doping and electron-beam (e-beam) treatment on photocatalytic performance were evaluated. The electrospinning process can fabricate nanoscale samples simply and inexpensively and is widely used due to its high yield [33,34]. TiO_2_ in nanofiber form has a high aspect ratio and good mechanical properties, which can improve photocatalytic performance [35,36]. Copper (Cu) is abundant in nature and inexpensive. In addition to its metallic form, both Cu(I) and Cu(II) species (including Cu_2_O and CuO) act as electron mediators, enhancing charge transfer and broadening the absorption spectrum into the long-wavelength region [37,38]. Therefore, copper doping can be an economical method to improve the efficiency of TiO_2_ photocatalysts. Furthermore, high-energy e-beam treatment can induce changes in crystal field energy, bonding states, and electronic structures, leading to greater enhancement in photocatalytic performance [39,40]. In this study, photocatalytic performance was evaluated through photodegradation tests using methylene blue (MB) solution. An MB photodegradation test was performed using samples with different molar ratios of copper to confirm the dopant concentration that optimizes photocatalytic performance. Subsequently, e-beams at different radiation doses were irradiated to samples with the optimal dopant concentration, and the MB photodegradation test was repeated. It was proven that photolysis efficiency increases as the electron beam irradiation dose increases. These results show that photocatalytic performance is improved by transition metal doping and high-energy e-beam treatment.

## 2. Results and Discussion

### 2.1. FE-SEM

**Figure 1:**
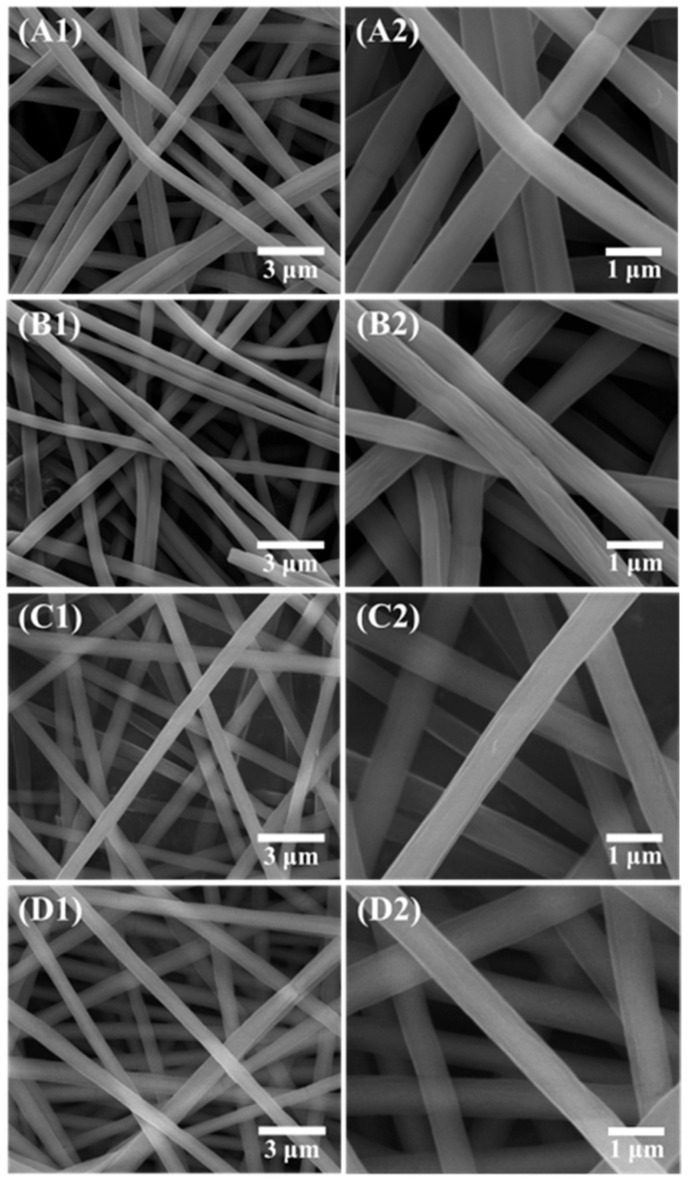
FE-SEM images of as-spun bare TiO_2_ and Cu-doped TiO_2_ at low and high magnification: (**A1**,**A2**) TNF0; (**B1**,**B2**) TNF1; (**C1**,**C2**) TNF2; (**D1**,**D2**) TNF3.

**Figure 2:**
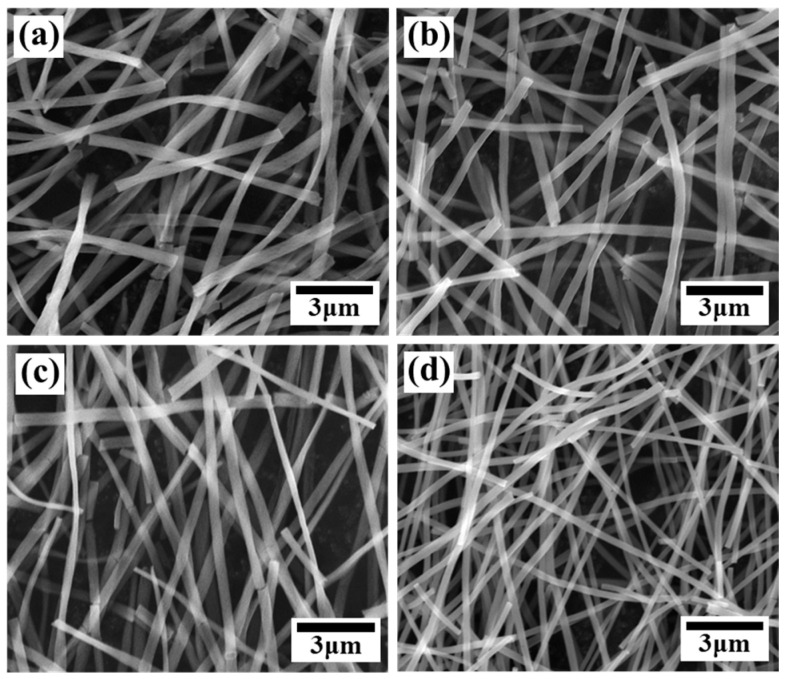
FE-SEM images of calcined bare TiO_2_ and Cu-doped TiO_2_: (**a**) TNF0; (**b**) TNF1; (**c**) TNF2; (**d**) TNF3.

**Figure 3:**
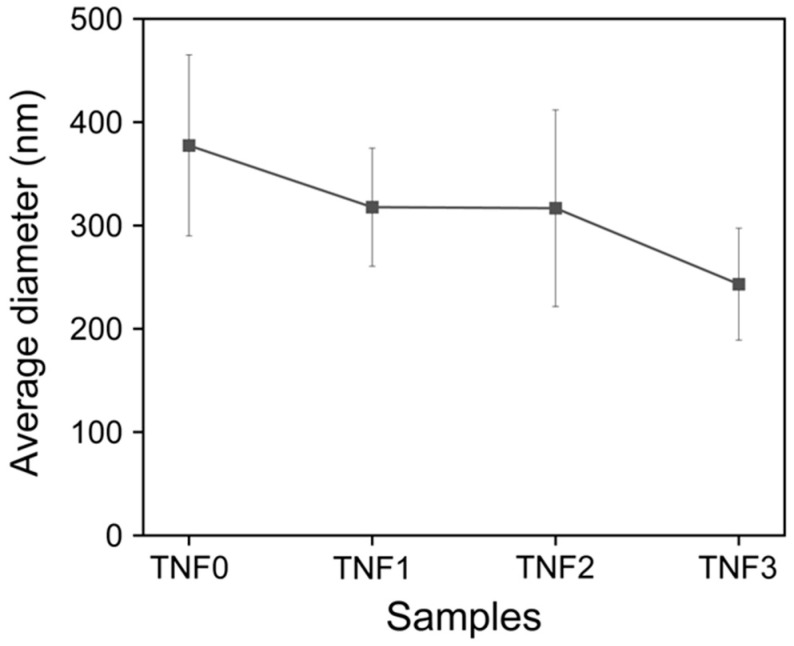
Average diameter graph of TNFs after calcination.

FE-SEM images were obtained to observe the microstructure of TNFs fabricated via electrospinning. Figure 1 shows the as-spun bare and Cu-doped TNFs, while Figure 2 shows the TNFs after calcination. As can be seen in Figure 1 and Figure 2, even after the removal of the PVP matrix through calcination, the nanofibers retained a homogeneous and continuous surface and morphology. The average diameter of the TNFs was determined by measuring at least 200 diameters from each sample, using the open-source software Image-J (ver. 1.8.0). The average diameters of the calcined TNFs were measured to be 377.52 nm, 317.71 nm, 316.77 nm, and 243.19 nm, respectively, as shown in Figure 3. As the Cu content increased, the average diameter of the TNFs decreased. Numerous studies have indicated that a reduction in fiber diameter may lead to an increased specific surface area, potentially enhancing photocatalytic efficiency [41,42,43].

**Figure 4:**
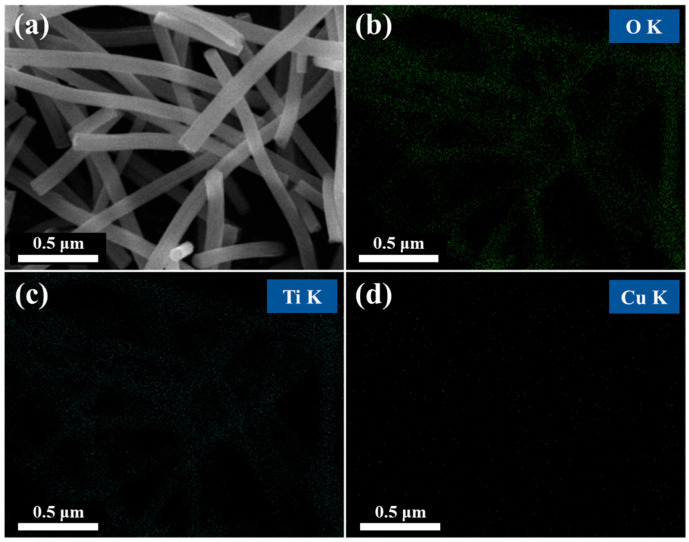
FE-SEM images of calcined TNF2 (**a**) and observed elemental distribution mapping of O (**b**), Ti (**c**), and Cu (**d**) in Cu-doped TiO_2_ nanocomposite sample TNF2.

Figure 4 presents the results of the Energy-dispersive X-ray spectroscopy (EDS) mapping of TNF2. After calcination, the presence of Ti, O, and Cu in the fibers was confirmed, and it was observed that the Cu signal was evenly distributed throughout the TiO_2_ nanofibers.

### 2.2. Photocatalytic Efficiency Depending on Cu Content

**Figure 5:**
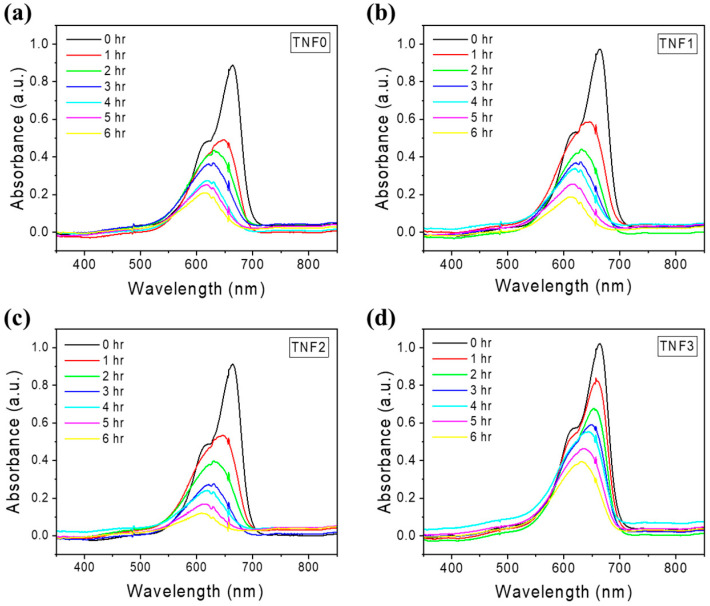
UV-Vis absorption spectra of photodegradation of methylene blue by bare TiO_2_ and Cu-doped TiO_2_: (**a**) TNF0; (**b**) TNF1; (**c**) TNF2; (**d**) TNF3.

**Figure 6:**
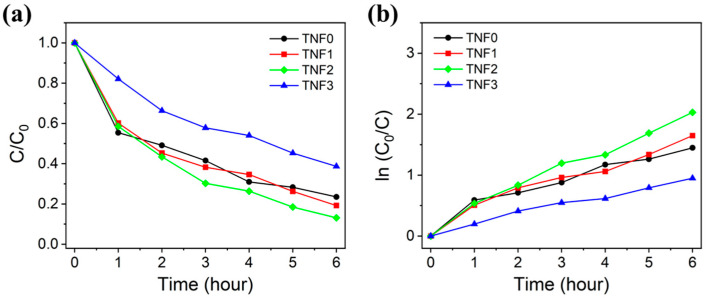
Methylene blue degradation curves (**a**) and kinetic curves (**b**) of bare TiO_2_ and Cu-doped TiO_2_.

The photocatalytic activity according to the Cu content was evaluated by the degradation of methylene blue (MB) in an aqueous solution. Figure 5 shows the absorption spectrum of the MB aqueous solution, sampled every hour during the photocatalytic reaction. A weakening absorption peak over time indicates the photodegradation of MB molecules by the TNFs. The maximum absorbance of MB was designated as the C value to calculate normalized values (C/C_0_) and ln(C_0_/C). The C value considers the fact that the maximum absorption wavelength is shifted to a shorter wavelength due to *N*-demethylation of methylene blue during the reaction process [44,45]. The changes in C/C_0_ and ln(C_0_/C) values over time are shown in Figure 6. The MB dye degradation rate was determined using the following Equation (1):

(1) $$Removal\left(\%\right)=\frac{C_{0}-C_{x}}{C_{0}}\times 100$$

C_0_ is the initial concentration of the MB aqueous solution and C_x_ is the concentration at time t.

As can be seen in Table 1, TNF2 was confirmed to have the highest photocatalytic activity. The addition of Cu was observed to improve the photodegradation rate. Many studies suggest that transition metals and metal doping can reduce the band gap of TiO_2_ and induce oxygen vacancies, thereby improving photocatalytic efficiency [46,47,48,49,50]. CuO and Cu_2_O have a lower Fermi level than TiO_2_, which leads to the transfer of excited electrons from TiO_2_ to CuO and Cu_2_O [51]. Cu^2+^ ions form Cu^+^ ions while acting as traps to capture photoexcited electrons of TiO_2_ (Equation (2)), and this electron trapping is effective in reducing the electron–hole recombination rate [51,52,53,54,55,56,57]. Cu^+^ ions can accelerate interfacial electron transfer by transferring electrons to oxygen adsorbed on the catalyst surface [56,57,58]. Equation (3) is the related reaction equation:

(2) $${Cu}^{2+}+e^{-}\to {Cu}^{+}$$

(3) $${{Cu}^{+}+O_{ads}\to {Cu}^{2+}+O}_{ads}^{-}$$

However, TNF3 showed lower photocatalytic activity than TNF0, which was undoped with Cu. This observation can be attributed to research findings indicating that photocatalytic activity decreases when the concentration of metal or transition metal doping exceeds a certain threshold [26,51,59,60]. Excessive oxygen vacancies and Cu species can act as recombination centers for photoexcited electron–hole pairs [51,61]. Another cause is CuO deposition on the TiO_2_ surface [51,61]. Therefore, an excessive dopant amount can reduce photocatalytic activity, and photocatalytic efficiency is greatly dependent on the concentration of the dopant.

### 2.3. XRD Analysis

**Figure 7:**
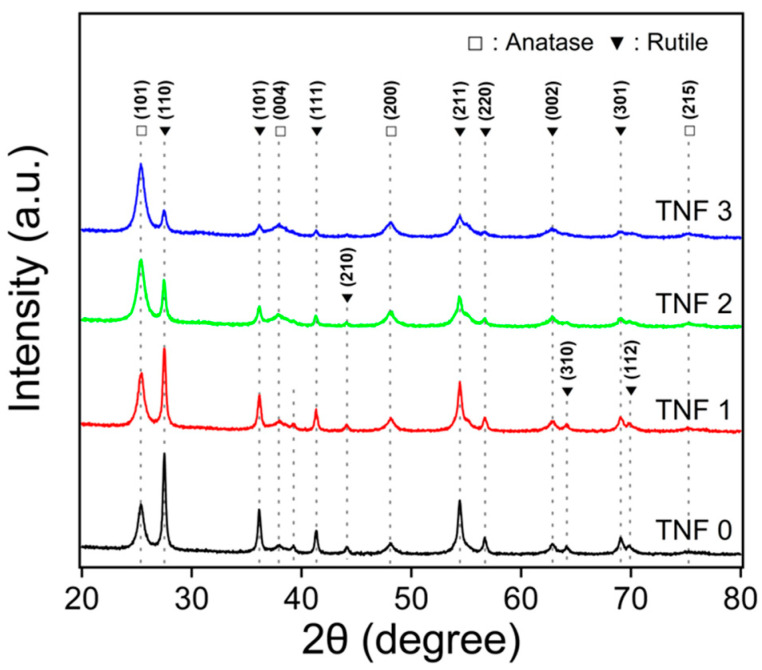
XRD patterns of bare TiO_2_ and Cu-doped TiO_2_.

Figure 7 shows the XRD diffraction pattern of Cu-doped TNFs calcined at 400 °C for 3 h. All samples exhibited the anatase phase of titanium dioxide and the rutile phase of titanium dioxide. The diffraction peaks at 2θ = 25.4°, 38.0°, 48.1°, and 75.2° correspond to the (101), (004), (200), (215) peaks, indicating the anatase phase of TiO_2_. Peaks at 27.5°, 36.2°, 41.3°, 44.1°, 54.4°, 56.7°, 62.8°, 64.1°, 69.1°, and 69.9° diffraction peaks are indexed with the (110), (101), (111), (210), (211), (220), (002), (310), (301), and (112) peaks, indicating the rutile phase of TiO_2_ [62]. In TNF2, the (310) and (112) peaks indicating the rutile phase were not observed, and in TNF3, the (210) peak was also not observed. Additionally, as the copper content increased, the peak intensity for the rutile phase decreased. Consequently, it was deduced that with an increase in Cu content, the proportion of the anatase phase became more dominant. The phase ratio of anatase and rutile for each sample are detailed in Table 2. At no concentration were crystalline phases corresponding to Cu, CuO, or Cu_2_O observed, suggesting that the Cu ions might be uniformly dispersed or that the amount of CuO or Cu_2_O was too minimal to detect.

### 2.4. Photocatalytic Efficiency According to E-Beam Irradiation

**Figure 8:**
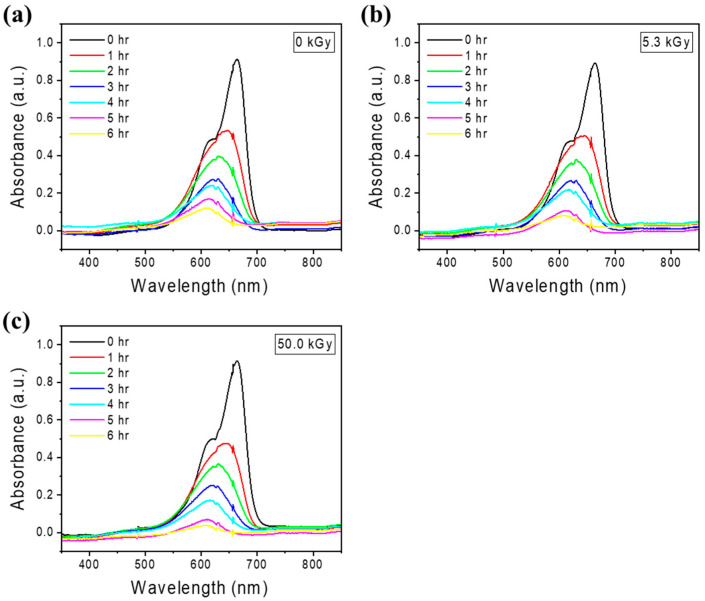
UV-Vis absorption spectra of photodegradation of methylene blue by unirradiated TiO_2_ and e-beam-treated TiO_2_: (**a**) 0 kGy; (**b**) 5.3 kGy; (**c**) 50 kGy.

**Figure 9:**
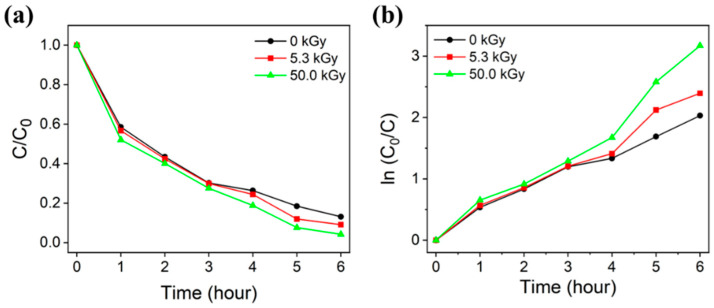
Methylene blue degradation curves (**a**) and kinetic curves (**b**) of unirradiated TNFs and e-beam treated TNFs.

The photocatalytic efficiency according to e-beam irradiation was evaluated using the same method and conditions as those used to evaluate photocatalytic efficiency according to Cu content. Samples were prepared by e-beam irradiation on TNF2, which showed the highest photodegradation rate in the evaluation of photocatalytic efficiency according to Cu content. A comparative analysis of the photodegradation rates of MB in aqueous solutions was conducted among samples not subjected to e-beam irradiation and those treated with 5.3 kGy and 50 kGy e-beam doses. It was observed that photodegradation efficiency increased with increased e-beam irradiation doses. Figure 8 shows the absorption spectrum of the MB aqueous solution sampled every hour during the photocatalytic reaction, and Figure 9 shows the C/C_0_ and ln(C/C_0_) values over time. The irradiation of e-beam significantly improved the efficiency and rate of photodegradation of MB by TNFs. As can be seen in Table 3, the sample irradiated with 50.0 kGy of e-beam photodegraded up to 95.80% after 6 h.

### 2.5. Raman Spectroscopy

**Figure 10:**
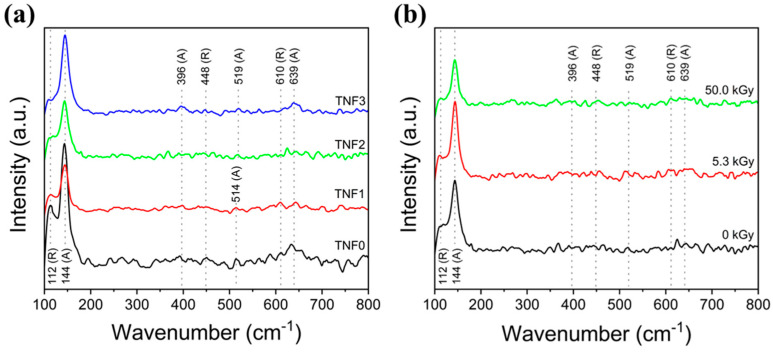
Raman spectra of TNFs: (**a**) bare TiO_2_ and Cu-doped TiO_2_; (**b**) unirradiated TiO_2_ and e-beam-treated TiO_2_.

Figure 10 shows the Raman spectrum of Cu-doped TNFs (a) and the Raman spectrum following e-beam treatment (b). All samples showed similar spectra, and the fingerprints closely matched the XRD patterns. The intense peak observed at 144 cm^−1^, along with Raman bands around 396, 514, 519, and 639 cm^−1^, correspond to the symmetry species E_g_, B_1g_, A_1g_, B_1g_, and E_g_, respectively. These Raman active modes are characteristic of anatase-TiO_2_ [63,64]. Also, the bands at 112, 143 (superimposed with the 144 cm^−1^ band by the anatase phase), 448, and 610 cm^−1^ correspond to symmetry species B_1g_, B_1g_, E_g_, and A_1g_, respectively, and are attributed to the rutile-TiO_2_ [63,64]. In Raman active vibrational modes, the E_g_ mode mainly corresponds to the symmetric stretching vibration of O-Ti-O, the B_1g_ mode is caused by the symmetric bending vibration of O-Ti-O, and the A_1g_ mode is caused by the anti-symmetric bending vibration of O-Ti-O [65,66].

### 2.6. XPS Analysis

**Figure 11:**
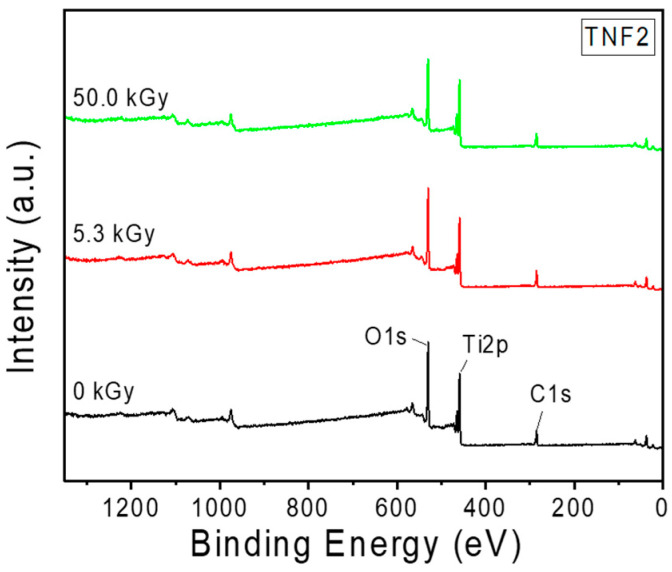
XPS spectra of unirradiated TiO_2_ and e-beam-treated TiO_2_.

**Figure 12:**
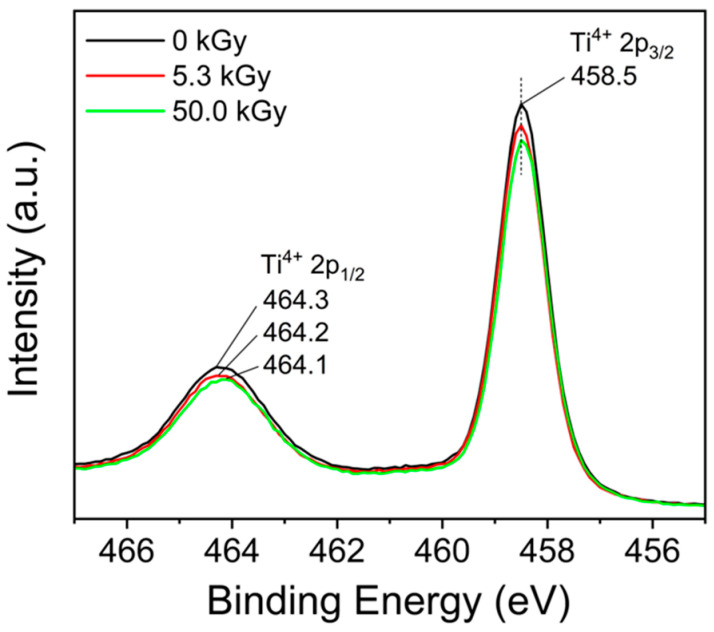
XPS spectra of Ti 2p on the surface of unirradiated TiO_2_ and e-beam-treated TiO_2_.

For surface characterization of e-beam treated and untreated TNFs samples, the X-ray spectroscopy (XPS) method was used. Figure 11 shows the XPS spectra of samples without e-beam irradiation and those irradiated with 5.3 kGy and 50.0 kGy, respectively. Figure 12 shows the Ti 2p peak for the three samples. Among the two peaks, the one with lower intensity corresponds to Ti^4+^ 2p_1/2_, and the one with relatively higher intensity corresponds to Ti^4+^ 2p_3/2_. As e-beam irradiation was performed, the height of the two peaks for Ti^4+^ 2p decreased, and the binding energy of Ti^4+^ 2p_3/2_ was the same at 458.5 eV, but the binding energy of Ti^4+^ 2p_1/2_ was 464.3, 464.2, and 464.1 eV, respectively, showing a tendency to decrease as the e-beam irradiation amount increased. This shift may be attributed to the growing influence of the Ti^3+^ 2p peak on the Ti^4+^ 2p peak. These peak shifts can be considered as a result of ionization, occurring when high-energy electron beams irradiate TiO_2_ nanofibers, causing some Ti^4+^ ions to capture electrons and convert into Ti^3+^ states. This change in the bonding state can promote the trapping of photo-excited charge carriers, thereby delaying recombination and enhancing the redox reactions on the surface, contributing to an improvement in photocatalytic performance. [67,68,69]. Additionally, high-energy e-beam irradiation creates oxygen vacancies in the surface layer and boosts the production of oxidizing agents such as hydroxyl radicals (•OH) and reactive oxygen species (•O_2_), thereby enhancing the adsorption and decomposition of pollutants [70,71,72]. Equations (2)–(5) represent the previously mentioned chemical reactions:

(4) $${TiO}_{2}+h\nu \to h^{+}+e^{-}$$

(5) $${Ti}^{4+}+e^{-}\to {Ti}^{3+}$$

(6) $$O_{2}+e^{-}\to {•O}_{2}^{-}$$

(7) $${H_{2}O+h^{+}\to •OH+OH}^{+}$$

## 3. Materials and Methods

### 3.1. Materials

Polyvinylpyrrolidone (PVP) (M.W. 1,300,000) was purchased from Alfa Aesar Korea Co., Ltd. (Incheon, Republic of Korea). Titanium (IV) isopropoxide (TTIP) (≥98.0%), Acetylacetone (ACAC) (≥99.0%), and Copper (II) acetate monohydrate (≥98.0%) were purchased from Junsei Co., Ltd. (Tokyo, Japan). Ethyl alcohol (EtOH) (anhydrous, ≥99.9%) was purchased from Samchun (Seoul, Republic of Korea). Methylene blue (MB) was purchased from Sigma-Aldrich Co., Ltd. (St. Louis, MO, USA).

### 3.2. Fabrication of Cu-Doped and Bare TiO_2_ Nanofibers

The precursor solutions for the electrospinning of TNFs were prepared with varying contents of Cu acetate, and the composition of each solution is shown in Table 4. The Cu content was adjusted to achieve molar ratios of 0, 0.02, 0.05, and 0.1% with titanium. In the first beaker, 40 g of PVP and the designed content of Cu acetate were added to 250 g of EtOH and stirred for 24 h. Subsequently, 50 g of ethanol, ACAC, and TTIP were added to another beaker and stirred until the solution became a homogenous, transparent yellow. The two solutions were then mixed and stirred at room temperature for an additional 24 h.

**Figure 13:**
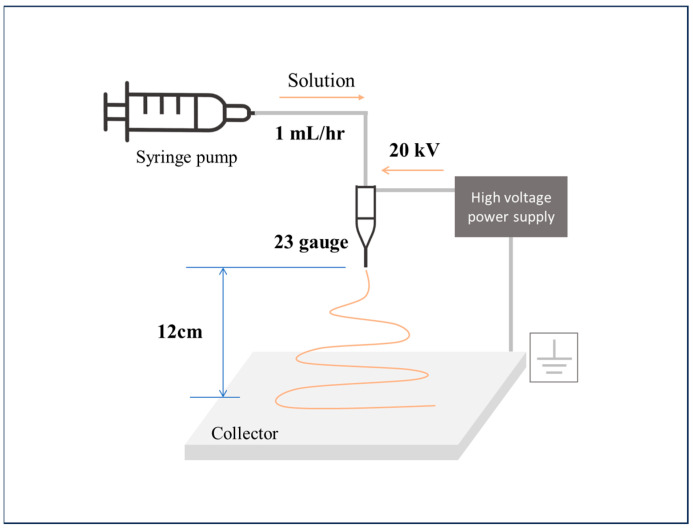
Schematic diagram of electrospinning process.

The precursor solution was fabricated and collected in nanofiber form using electrospinning, and Figure 13 shows a schematic of the electrospinning process. The electrospinning parameters were as follows: a needle tip-to-collector distance of 12 cm, a flow rate of 1 mL/h, and a 23-gauge needle tip diameter. A high voltage of 20 kV was applied through a power supply. Each fabricated sample was first dried in an oven at 60 °C and then calcined by heating in a box furnace to 400 °C for 3 h at a rate of 5 °C/min.

### 3.3. Characterization

The surface and diameter of the TiO_2_ nanofiber samples were characterized using a field-emission scanning electron microscope (FE-SEM). The crystal structure and crystallinity of the TiO_2_ sample were analyzed using an X-ray diffractometer (XRD) with Cu Kα line radiation. Raman spectroscopy was used to analyze the chemical structure of the sample, using a laser with a 532 nm wavelength. UV-visible spectrophotometer (UV-Vis) measurements were recorded in the range of 350–850 nm. The X-ray Photoelectron Spectroscopy (XPS) method was employed to analyze the surface properties of the e-beam irradiated samples. A monochromatic X-ray source Al-Kα was used, and all binding energies (BEs) were calibrated by the BE (284.8 eV) of C 1 s.

### 3.4. Photocatalytic MB Degradation

To evaluate the photocatalytic efficiency of both bare TiO_2_ and Cu-doped TiO_2_, the degradation test of methylene blue (MB) dye was performed. An aqueous MB solution was prepared at a concentration of 5 mg/L by dissolving MB powder in distilled water. A mixture of 10 mL of distilled water and 0.1 g of the TiO_2_ catalyst was added in a quartz beaker and stirred for 30 min in darkness. Subsequently, 100 mL of the MB aqueous solution was added, followed by its exposure to UV light with a 365 nm wavelength for 6 h. The UV lamp (VL-6.LM, Vilber Lourmat, Eberhardzell, Germany) was positioned 10 cm from the beaker, with a stirring speed of 220 rpm. Throughout the photodegradation test, approximately 5 mL of samples were extracted every hour using a syringe. The TiO_2_ catalyst was filtered from these samples using a syringe filter (PTFE, 0.2 µm, CHMLAB group, Barcelona, Spain), and the filtrate was then placed into a cuvette. The photocatalytic efficiency was determined by measuring the absorbance between 350 and 850 nm using a UV-Vis spectrophotometer.

### 3.5. E-Beam Treatment Test

To investigate the effect of e-beam irradiation on photocatalyst efficiency, Cu-doped TiO_2_, which exhibited the highest photodegradation efficiency, was irradiated with an e-beam at doses of approximately 5.3 kGy and 50.0 kGy. The TNFs, pulverized into powder and contained in a PE bag, were exposed to the e-beam using an electron accelerator (ELV-8 type), as per the parameters shown in Table 5. Then, a photocatalytic MB degradation test using UV-Vis spectroscopy was performed to measure the absorbance of the filtered MB aqueous solution, comparing the photodegradation efficiency between the samples treated with and without electron beam irradiation.

## 4. Conclusions

TiO_2_ nanofibers, fabricated by controlling the amount of Cu added, were irradiated with e-beams and compared in terms of their photocatalytic properties. An appropriate amount of Cu doping and high-dose e-beam irradiation significantly improved the decomposition rate of MB dye on the TiO_2_ photocatalyst. Cu doping reduced the average diameter of TiO_2_ nanofibers and increased the anatase-TiO_2_ phase fraction, but the photocatalytic efficiency of samples doped with more than a certain amount of Cu decreased. This decrease is attributed either to the generation of excessive oxygen vacancies or to the Cu species acting as recombination centers for photoinduced electrons and holes. Therefore, it was confirmed that an optimal concentration exists for performance improvement via Cu doping. As the e-beam irradiation dose increased, the photocatalytic efficiency increased too. Irradiation with an e-beam creates oxygen vacancies on the surface and increases the generation of Ti^3+^ ions, which can serve as a medium for interfacial charge transfer.

## Figures and Tables

**Table 1:** MB dye degradation rate (%) after 6 h according to Cu content.

TNF0	TNF1	TNF2	TNF3
76.48	80.75	86.87	61.30

**Table 2:** The phase ratio of each sample depending on the Cu content.

Sample	Anatase (%)	Rutile (%)
TNF0	20	80
TNF1	26	74
TNF2	41	59
TNF3	59	41

**Table 3:** MB dye degradation rate (%) after 6 h according to e-beam treatment.

0 kGy	5.3 kGy	50.0 kGy
86.87	90.87	95.80

**Table 4:** The composition of precursor solutions (unit: g).

Sample	PVP	EtOH	Cu Acetate	TTIP	ACAC	Molar Ratio Cu/Ti (%)
TNF0	40	300	0	50	50	0
TNF1	40	300	0.007	50	50	0.02
TNF2	40	300	0.018	50	50	0.05
TNF3	40	300	0.035	50	50	0.10

**Table 5:** The E-beam irradiation conditions for each sample.

Acceleration Voltage (MeV)	Beam Current (mA)	Processing Number	Radiation Exposure (kGy)
0	0	0	0
0.8	4	1	5.3
1.2	5	5	50.0

## Data Availability

Data are contained within the article.
